# Domestic

**Published:** 1840-07-18

**Authors:** 


### DOMESTIC.

*Medical Department of Pennsylvania College.*—The annual announcement for the current year presents a list of twenty-eight graduates, who received the degree of Doctor of Medicine in March last.

*Medical Institute of Louisville.*—Professor Samuel D. Gross has been added to the faculty of this institution, for the chair of surgery. Professor Gross's reputation as a writer and teacher, makes him a valuable acquisition. Two hundred and four pupils attended the late course at Louisville, thirty-nine of whom graduated.

*Medical Institution of Geneva College.*—The three vacant professorships in this institution have been filled by Professors Hadley, Delamater, and Hamilton. Nineteen graduates are announced for the past session.

*Medical Graduates for* 1840 *in the United States.*—The University of Pennsylvania, 163; Medical College of South Carolina, 65; Transylvania University, 60; Jefferson Medical College, 57; Medical Institute of Louisville, 39; Medical Department of Pennsylvania College, 28; Medical Institution of Geneva College, 19; Washington University of Baltimore, 19; University of Maryland, 14.

---

*Case of Forcible Removal of the Uterus and its Appendages, after the expulsion of the Fœtus.*—We are indebted to Dr. Drane, of this city, for the following particulars of this most extraordinary case. A woman residing in Oldham county, in this state, was attended by a midwife in her fourth or fifth confinement. Shortly after the birth of the child, the midwife applied herself to the task of removing the placenta, and seizing hold of the os tincæ, which was taken for the placenta, she applied such extractive force as to lacerate the vaginal and ligamentous attachments of the uterus, and bring away the entire organ with the remnants of its ligaments, the fallopian tubes and ovaria. Very little hæmorrhage followed this rude operation; but the patient being alarmingly prostrated by the violence she had suffered, Dr. Ballard, of Westport, was summoned to her assistance. When the doctor arrived and inquired concerning the delivery, he was informed by the midwife that the patient was cleared, and his attention was directed to a vessel containing the supposed after-birth, as evidence that she had performed her whole duty. He was surprised and alarmed for the safety of his patient to find on examination that it was the uterus and its appendages, which were deposited in the vessel, and on making a section of the uterus, the placenta was found enclosed in its cavity.

Dr. Drane did not see the patient, nor is he informed as to the history of the case after the accident: he only knows that, without any very serious consequences, the woman recovered perfectly; that she is at this time alive and in good health, and has borne no children since her mutilation. He had more than one opportunity of examining the parts, preserved by Dr. Ballard, and, perhaps, still in his possession, and he assures us unequivocally that they comprise the uterus, containing the placenta, the tubes, ovaria, and portions of the uterine ligaments. We have such confidence in his good faith and competency to decide on such questions, that ocular demonstration could not make us more certain of the fact.

This case, as far as our recollection serves us, is unique in the annals of obstetric medicine. There are well authenticated instances of inversion of the uterus following delivery, wherein the organ has been ignorantly torn away, or has been lost by inflammation and sloughing. In the Medico-Chirurgical Review, vol. xxiv., p. 482, a case of this kind is recorded, which had been considered of sufficient interest to be published in pamphlet form by Mr. Cooke. As this able periodical may not be in the hands of all our readers, or this case may have been overlooked by them, we shall make no apology for extracting the following summary of it, premising the single remark, that as the placenta was naturally and promptly expelled, and the inversion took place twenty-four hours after delivery, apparently in consequence of the woman's rising to urinate, and as when the midwife was summoned, although only a few hours had elapsed, the uterus was detached and removed merely by her lifting it gently with her hands, it is difficult to account for its separation, unless we suppose that the patient herself had inadvertently attempted to tear it away.

"At 4 A. M. of the 22d of May, 1835, a midwife of Coventry was sent for to a woman in labour. On her arrival, she found the woman, who had already been in labour forty-eight hours, upon her knees, and insisting upon being delivered in that position, as being the custom of her country, (Ireland.)

"With some difficulty she induced her to lie upon the bed, in order, by an examination of the parts, to ascertain how far the labour had advanced. Upon this being done, the os tincæ was found dilated to about the size of half-a-crown; the child's head presenting as usual. Every thing went on well, and the woman was delivered at 7 o'clock the same evening of a living child. The placenta followed whole in a quarter of an hour, being expelled by a pain.

"No hæmorrhage whatever ensued at the time, although a considerable quantity of blood was lost during the night. The after-pains were trifling; and when the midwife visited her the next morning, she appeared in a very satisfactory state. So well, indeed, did she feel, that notwithstanding the strict injunctions of the midwife to the contrary, she partook plentifully of animal food immediately after her departure. At 4 A. M. of the 24th the midwife was again hastily summoned. She ascertained the following particulars: that the woman had risen during the night, and had gone into an adjoining room to make water; that whilst there she had, by her screams alarmed her husband, who called in some of the neighbours; and upon entering the apartment, they found the woman seated on a stool, before the fire, with a vessel of warm water in front of her, and a large substance, which they compared to a child's head and neck, lying between her thighs, supported by her hands. They then sent for the midwife, believing it to be a case of twins, and being greatly alarmed at the quantity of blood she had lost, the hæmorrhage having been profuse. She was now lying on the bed, pale from the loss of blood, which had been excessive. On examination, the midwife discovered a hard substance, lying on the bed, loosely connected to the vagina by a shred of membrane only. This substance she immediately recognised as the uterus, and lifting it gently, removed it without difficulty or effort, and placed it in a wooden bowl. The hæmorrhage then ceased, and the midwife, after a short delay, left the house, taking the uterus to our author's father. He found that it really was an uterus inverted. The only part absent was the left ovary. Mr. Cooke, senior, visited the woman at 11 A. M., and found her completely exhausted from the hæmorrhage, which by this time had ceased; she was extremely restless and agitated, constantly throwing her arms about; pulse scarcely perceptible. Upon inquiry he found she had passed some urine shortly after the loss of the uterus, as also between 9 and 10 the following morning, (Saturday.) He was also informed that her bowels had not been relieved for a period of at least nine days before delivery, except a mass of hardened fæcal matter, which was discharged with the last pain in the labour. The woman did not much complain of any sensation like bearing down, nor of any substance lying in the vagina. She did not appear to suffer much from pain; neither was there much distention of the abdomen, nor was there then or at any time during the progress of the case any thing amounting to more than a slight degree of tenderness, which, however, was hardly noticed except upon pressure. As there was no protrusion of the pelvic or abdominal viscera, and as there were no urgent symptoms, Mr. C. determined not to incur any risk by instituting an examination per vaginam. He merely enjoined quietude and the horizontal posture, with a light farinaceous diet.

"In the afternoon the pulse rose to one hundred and forty. The patient passed her urine freely during the night, and next day presented no unfavourable symptoms. In short, no material symptoms ensued, and she perfectly recovered without any surgical, and scarcely any medical treatment."

This case is interesting to the physiologist as well as to the accoucheur, inasmuch as it presented a striking illustration of the close sympathy which exists between the uterus and mammæ, which might have been observed, we doubt not, in Dr. Ballard's case.

We are informed that "previous to her confinement, milk was secreted in considerable quantity; but immediately after the loss of the uterus, this secretion, together with that of the lochia, was arrested. Notwithstanding this, and in spite of the remonstrances which were made to her on the subject, she persisted in applying the child to the breast, which induced considerable pain and hardness of the right mamma, attended with a good deal of febrile excitement. These symptoms subsided upon the exciting cause being removed. When her health had been in some degree re-established, she again gave the child the breast, and persevered in doing so during several weeks, until finding that she had no milk she finally desisted."

It is furthermore interesting, because it shows that notwithstanding the preservation of one ovary, all sexual desires and feelings were entirely wanting, although sexual intercourse has repeatedly been had with her husband, no mechanical obstruction whatever existing. This is contrary to what obtains with the other sex; for ridglings, it is well known, retain their sexual propensities.—*Western Journal of Medicine and Surgery.*

